# The Effects of Chlorhexidine Dressing on Health Care-Associated Infection in Hospitalized Patients: A Meta-Analysis

**Published:** 2019-05

**Authors:** Hou-Xing WANG, Shu-Yuan XIE, Hao WANG, Hao-Kai CHU

**Affiliations:** Department of Emergency Intensive Care Unit, Yinzhou Second Hospital, Ningbo, China

**Keywords:** Chlorhexidine dressing, Catheter-related bloodstream infections, Randomized controlled trials

## Abstract

**Background:**

To assess the effects of chlorhexidine dressing on health care-associated infection in hospitalized patients.

**Methods:**

We searched for English-language published randomized controlled trials (RCTs) in Cochrane Library, EMBASE and PubMed between January 1998 and January 2018. We used meta-analysis to calculate the risk ratios (RRs) and 95% confidence intervals (CIs) of the data, and using the *I*^2^ assessment to summarize the heterogeneity of RCTs and the funnel plot and Egger regression test to evaluate publication bias.

**Results:**

A total of 13 RCTs were included in our meta-analysis, including 7555 patients and 11,931 catheters. The effects of chlorhexidine dressing on the incidence of catheter-related bloodstream infections (CRBSIs) were reported in 13 RCTs, and the incidence of CRBSIs were 1.3% (80/6160) in the chlorhexidine group and 2.5% (145/5771) in the control group. We used a forest plot to determine the risk ratio (RR) of chlorhexidine dressing on the incidence of CRBSIs, and our results showed that chlorhexidine dressing significantly reduced the incidence of CRBSIs (RR 0.55, 95% CI 0.39–0.77, *P*<0.001). Moreover, we also analyzed the effects of chlorhexidine dressing on the incidence of catheter colonization and catheter-related infections (CRIs), and our forest plot results showed that chlorhexidine dressing significantly reduced the incidence of catheter colonization (RR 0.52, 95% CI 0.40–0.67, *P*<0.001) and the incidence of CRIs (RR 0.43, 95% CI 0.28–0.66, *P*<0.001) in hospitalized patients.

**Conclusion:**

The use of chlorhexidine dressings for hospitalized patients significantly reduce the incidence of CRBSIs, catheter colonization and CRIs.

## Introduction

Central venous catheters (CVCs) are an important source of bloodstream infections (BSIs) in hospitalized critically ill patients and are closely related to patients’ mortality (1). During the hospitalization, patients complicated with catheter-related bloodstream infections (CRBSIs) and/or catheter-related infection (CRIs) caused their illness to worsen, the length of hospital stay was extended, and hospitalization expenses increased (2–4). According to data reported by the Centers for Disease Control and Prevention in US in 2009, the number of CRBSIs in the Intensive Care Unit (ICU) was 12,000–18,000, and the medical expenses generated per case were about $16,550, and the overall mortality rate was increased by 15%–25% (5).

At present, due to the limited number of antimicrobial drugs and the emergence of multi-drug resistance, the task of anti-infection is becoming more and more difficult. The Clinical Laboratory Standards Association has developed a standardized method for testing antimicrobial sensitivity, reliability and repeatability (6). The main mechanism of CRBSIs is the in vivo bloodstream contamination caused by the translocation of microorganisms through the skin of the catheter into the blood vessels (7). Therefore, blocking the pathway by which microorganisms invade the blood from the skin is an important method for reducing CRBSIs. Chlorhexidine has a broad spectrum of antibacterial activity against Gram-positive bacteria, Gram-negative bacteria, aerobic bacteria, anaerobic bacteria and fungi, and the use of chlorhexidine for skin disinfection in ICU patients reduces the spread of microbes and the incidence of CRBSIs (8).

In recent years, there has been increasing interest in using chlorhexidine to disinfect skin to reduce acquired infections in hospitalized patients. Chlorhexidine dressings reduce the incidence of CRBSIs (9–13), but some studies have the opposite result, do not support the use of chlorhexidine dressings (14–18). Therefore, in this study, we used a meta-analysis to determine the effects of chlorohexidine dressings on the incidence of CRBSIs, catheter colonization and CRIs in hospitalized patients.

## Methods

### Search Strategy

Under the guidance of librarians, we searched for published studies between January 1998 and January 2018 in three large databases worldwide, including Cochrane Library, EMBASE and PubMed. The keywords were used in the search include: “Chlorhexidine”, “dressing(s)”, “Catheter-related bloodstream infections”, “Catheter-related Infections”, “Central line-associated bloodstream infections” and “catheter colonization”. Inclusion criteria: 1.) The selected articles were all published in English; 2.) Randomized controlled trials (RCTs) published before January 2018; 3.) Hospitalized patients used chlorhexidine dressings; 4.) Access to detailed clinical data.

### Data Abstraction

We developed a standardized form for extracting all the data, and the two judges independently read the full text of the article and extracted the data. If there was a disagreement between the results or data extracted by the two senators, the third senator presided over the negotiation and discussion to resolve the differences. The data used by our study was limited to published results. The data extracted from each study included: authors of the article, time of publication, study population, department, chlorhexidine group and control group, clinical outcomes, related definitions, etc. The primary outcome was the correlation between chlorhexidine dressing and CRBSIs. The secondary outcome was the effects of chlorhexidine dressing on the incidence of catheter colonization and CRIs.

### Risk of Bias Assessment

We used the Cochrane bias risk tool to assess the risk of RCTs bias in each article. According to the methods, two authors independently make high, low or unclear material deviation risk judgments for each RCT (19). We used Review Manager 5.2 to assess the risk of bias in the included studies.

### Statistical Analysis

One author entered the obtained data into Review Manager 5.2 software, and another author verifies the accuracy of the input data. We used meta-analysis to calculate the risk ratios (RRs) and 95% confidence intervals (CIs) of the data, and using the *I*^2^ assessment to summarize the heterogeneity of RCTs. When *I*^2^> 50% or *P*≥0.10, the heterogeneity was considered significant (20), and we used the random-effects model. If the heterogeneity was not significant, we used a fixed-effects model. We used the Egger regression test and the funnel plot to evaluate publication bias (21). A *P* value <0.05 was considered statistically significant.

## Results

### Included studies

**Fig. 1:**
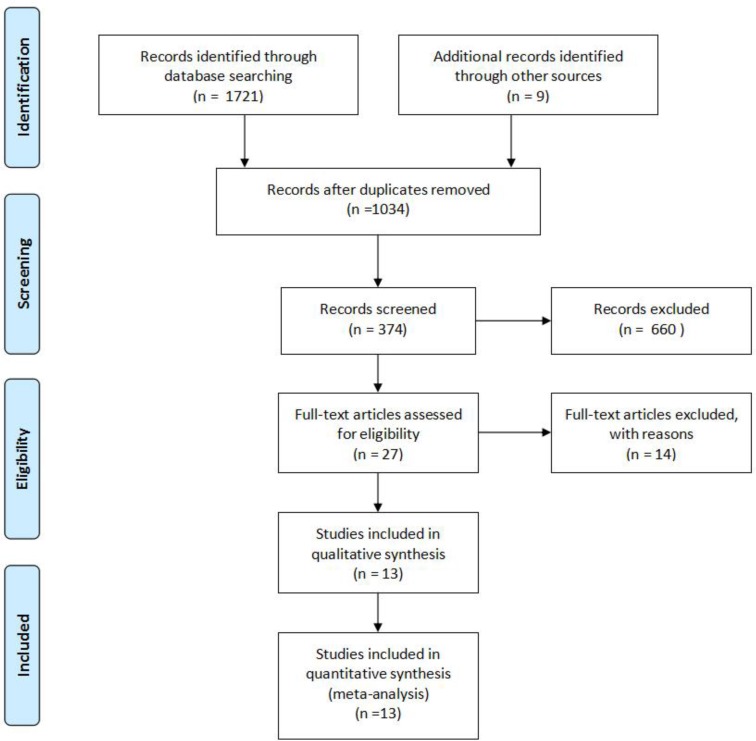
Flowchart for the study selection process

We searched a total of 1,034 documents in three large databases (Fig. 1.), including 105 from the Cochrane database, 136 from the Embase database, and 793 from the PubMed database. Overall, 660 articles were excluded because these documents did not meet the inclusion criteria, such as 518 articles were related to “chlorhexidine bathing”, 132 articles were “review” or “comment”, 9 articles were animal experiments, and 1 articles could not be searched for full text. The 27 full-text articles were fully reviewed, 11 articles were not RCT, 2 studies were incomplete, and one articles did not obtain the required data. Finally, a total of 13 RCTs were included in our meta-analysis (9–18, 22–24), 13 of which involved the relationship between chlorhexidine dressing and CRBSIs (8 studies in the ICU (9, 10, 12–15, 19–21, 23, 24) and 5 in the non-ICU (11, 16–18, 22)), 7 studies related to the effects of chlorhexidine dressing on catheter colonization (9, 10, 13–15, 23, 24), and 4 articles related to chlorhexidine dressing and CRIs correlation (10, 13, 15, 24).

### Trial Characteristics

The characteristics of the 13 RCTs were summarized in Table 1, which includes study time, population, department/setting, catheter type, skin disinfection method, chlorhexidine group and control group for each study. Among them, 4 RCTs were for children (9, 13, 18, 23), 2 RCTs were conducted by the same center at different time periods (10, 24), and 1 study did not provide a time interval (14). In addition, the relevant definitions and conclusions involved in each of the studies were summarized in Table 2.

**Table 1:** Characteristics of the included studies

***Study***	***Population***	***Setting***	***Catheter Type***	***Skin antiseptic***	***Intervention***	***Control***	***Duration***
Roberts BL et al. 1998 (14)	Adult patients requiring CVC during a 7 week period	ICU	CVCs	Chlorhexidine 0.5% in 70% alcohol	Chlorhexidine impregnated dressing	Occlusive dressing	NA
Garland JS et al. 2001 (9)	Neonates with CVC expected to remain in place a minimum of 48 hours	Neonatal ICU	CVCs	Intervention group: 70% alcohol scrub, Control group: 10% povidone iodine skin scrub	Chlorhexidine dressing	Polyurethane dressing	June 1994 to August 1997
Chambers ST et al. 2005 (22)	Adult patients undergoing chemotherapy	Haematology unit	CVCs	Alcohol povidone iodine 10%	Chlorhexidine dressings	No dressing	August 1998 to December 2001
Levy I et al. 2005 (23)	Pediatric patients requiring CVC for minimum of 48 hours	Pediatric cardiac ICU	CVCs	Chlorhexidine	Chlorhexidine gluconate impregnated sponge dressing	Polyurethane dressing	January 2002 to March 2003
Ruschulte H et al. 2009 (11)	Adults with hematologic or oncologic malignancy with catheter expected for minimum of 5 days	Haematology and oncology unit	CVCs	Alcohol spray	Chlorhexidine gluconate-impregnated wound dressing	Standard sterile transparent wound dressing	January 2004 to January 2006
Timsit JF et al. 2009 (10)	Adult patients requiring catheter minimum of 48 hours	ICU	CVCs, arterial catheter	4% aqueous povidoneiodine scrub solution followed by 5% povidoneiodine in 70% alcohol solution	Chlorhexidine gluconate–impregnated sponge dressing	Standard dressing	December 2006 to June 2008
Arvaniti K et al.2012 (15)	Adult patients requiring catheter at least 72 hours	ICU	CVCs	NA	Chlorhexidine gluconate–impregnated sponge dressing	Standard dressing	June 2006 to May 2008
Timsit JF et al. 2012 (24)	Adult patients expected to require catheter for at least 48 hours	ICU	CVCs	Alcoholpovidone or alcohol chlorhexidine	Chlohexidine-gel dressing	Standard dressing	May 2010 to July 2011
Scheithauer S et al. 2014 (12)	NA	A medical ICU and a cardiology ICU	CVLs	0.1% octenidine dihydrochloride and 2% 2-phenoxyethanol	Chlorhexidine-containing dressing	Standard dressing	November 2010 to may 2012
Düzkaya DS et al. 2016 (13)	Pediatric patients	Pediatric ICU	CVCs	10% povidone-iodine	2% Chlorhexidine impregnated dressing	Sterilized pad	December 2012 to January 2014
Biehl LM et al. 2016 (16)	Patients undergoing chemotherapy with an expected CVC use of ≥10 days	Hematology department	CVCs	Alcohol chlorhexidine	Chlorhexidine-containing dressing	Non-chlorhexidine control dressings	February 2012 to September 2014
Webster J et al. 2017 (17)	Hospital inpatients requiring a peripherally inserted central catheter	Tertiary referral hospital	PICCs	2% chlorhexidine gluconate in 70% isopropyl alcohol	Chlorhexidine gluconate dressing	Polyhexamethylene biguanide disc dressing	February 2016 to July 2016
Gerçeker GÖ et al. 2017 (18)	Pediatric hematology-oncology patients	Pediatric hematology unit	CVCs	Chlorhexidine gluconate	Chlorhexidine dressing	Advanced dressing	October 2014 to May 2015

CVC(s), central venous catheter(s); CVLs, central venous lines; PICCs, peripherally inserted central catheters; ICU, intensive care unit; NA, not applicable

**Table 2:** Outcomes from the included studies

***Study***	***Definitions of CRBSIs***	***Definition of catheter colonization***	***Definition of CRIs***	***Outcomes***	***Conclusion***
Roberts BL et al. 1998 (14)	Clinical infection with the same organism isolated from catheter tip and blood	Isolation of the same organism from CVCs tip and exit site, and the organism was not from an infection	NA	Incidence of CRBSIs, incidence of catheter colonization	No statistical difference
Garland JS et al. 2001 (9)	Clinical infection with same organism isolated from catheter tip and blood	Semi-quantitative catheter colony count >15 cfus	NA	Incidence of CRBSIs, incidence of catheter colonization	CRBSIs decreased
Chambers ST et al. 2005 (22)	Fever and positive blood cultures without alternative infection source, and catheter tip culture with >15 colonies of the same organism	NA	NA	Incidence of CRBSIs	Exit-site/tunnel infections decreased
Levy I et al. 2005 (23)	Bacteremia with isolation of the same organism from CVCs tip and blood	>15 cfus by the roll-plate technique, without signs of infection	NA	Incidence of CRBSIs, incidence of catheter colonization	Catheter colonization decreased
Ruschulte H et al. 2009 (11)	Clinical evidence of infection and time-to positivity method used with CVC and peripherally drawing blood cultures	NA	NA	Incidence of CRBSIs	CRBSIs decreased
Timsit JF et al. 2009 (10)	Clinical infection without alternative source and quantitative catheter tip culture isolating the same organism	Quantitative CVC tip culture ≥1000 cfus/mL	Catheter-related clinical sepsis without bloodstream infection and/or catheter related bloodstream infection	Incidence of CRBSIs, incidence of catheter colonization, incidence of CRIs	CRBSIs decreased
Arvaniti K et al.2012 (15)	Quantitative CVC tip culture with >1000 cfus/mL with systemic signs of sepsis	Quantitative CVC tip culture with >1000 cfus/mL and no systemic signs of sepsis	Positive quantitative culture of the tip plus clinical evidence of sepsis without additional sites of infection with the same microorganism	Incidence of CRBSIs, incidence of catheter colonization, incidence of CRIs	No statistical difference
Timsit JF et al. 2012 (24)	Correlation between peripheral blood culture and quantitative tip culture without other likely source	Quantitative CVC tip culture >1000 CFU/mL and no systemic signs of sepsis	Catheter-related clinical sepsis without bloodstream infection and/or catheter related bloodstream infection	Incidence of CRBSIs, incidence of catheter colonization, incidence of CRIs	CRIs decreased
Scheithauer S et al. 2014 (12)	NA	NA	NA	Incidence of CRBSIs	CRBSIs decreased
Düzkaya DS et al. 2016 (13)	>15 cfus in the catheter-end culture, and microorganisms in the 2 blood samples that have the same antibiotic resistance pattern as the microbes in the catheter end	>15 cfus in the catheter-end culture, without signs of infection	>15 cfus in the culture of the catheter end and fndings of inflammation at the catheter insertion site without blood-borne infection	Incidence of CRBSIs, incidence of catheter colonization, incidence of CRIs	CRBSIs decreased, Catheter colonization decreased
Biehl LM et al. 2016 (16)	According to the AGIHO-DGHO guidelines (2)	NA	NA	Incidence of CRBSIs	No statistical difference
Webster J et al. 2017 (17)	Bacteraemia or fungaemia obtained from a peripheral vein and taken while the PICC was in situ, or within 48 h of removal	NA	NA	Incidence of CRBSIs	No statistical difference
Gerçeker GÖ et al. 2017 (18)	According to the AGIHO-DGHO guidelines (2)	NA	NA	Incidence of CRBSIs,	No statistical difference

CVC(s), central venous catheter(s); CRBSIs, catheter-related bloodstream infections; CRIs, catheter-related infections; NA, not applicable; AGIHO-DGHO, the Infectious Diseases Working Party (AGIHO) of the German Society of Hematology and Medical Oncology (DGHO)

### Quality Assessment

**Fig. 2:**
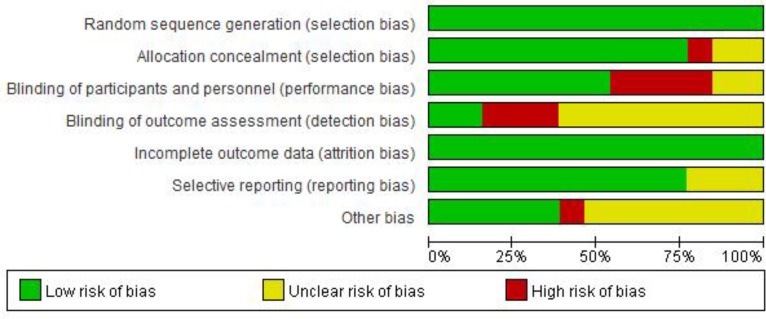
Risk of bias graph for the randomized controlled trials

We used Cochrane bias to assess selection bias or attribution bias in 13 RCTs. As shown in Figs. 2. and 3, because we did not retrieve the blinded evaluation of the study results, the risk of detection and performance bias in most studies was not clear. Three studies showed a high risk of bias due to lack of participants and personnel blinding (11, 12, 22).

**Fig. 3:**
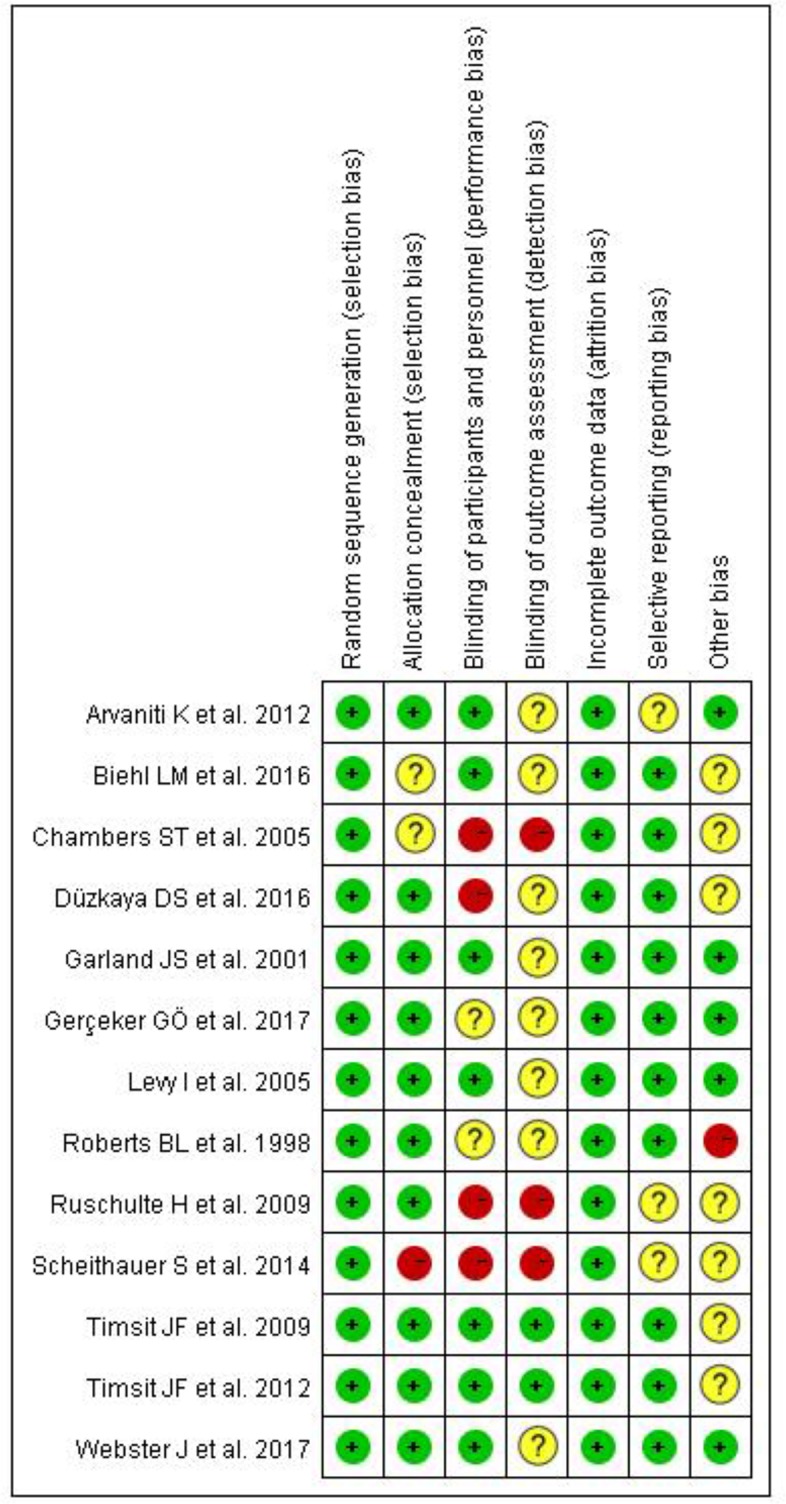
Risk of summary for the randomized controlled trials. “+” indicates a low risk of bias, “−” indicates a high risk of bias, and “?” indicates an unclear risk of bias

### Clinical outcomes

**Fig. 4:**
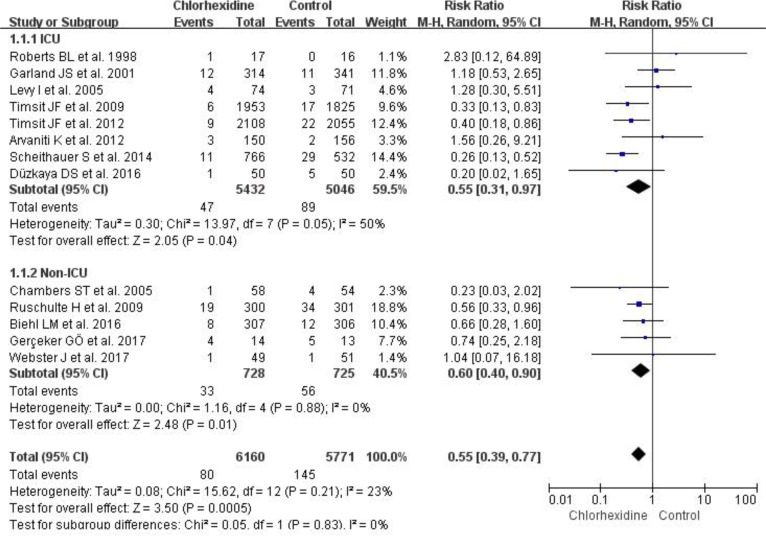
Forest plot of chlorhexidine dressing and control groups on the incidence of catheter-related bloodstream infections using a random-effects model. M-H indicates Mantel-Haenszel

A total of 7555 patients and 11,931 catheters were included in the 13 RCTs (9–18, 22–24), including 6,160 catheters in the chlorhexidine group and 5,771 catheters in the control group. The effects of chlorhexidine dressing on the incidence of CRBSIs were reported in 13 RCTs, and the incidence of CRBSIs was 1.3% (80/6160) in the chlorhexidine group and 2.5% (145/5771) in the control group, of which 5 studies indicated chlorhexidine dressing significantly reduced the incidence of CRBSIs (9–13). We used a forest plot to determine the risk ratio of chlorhexidine dressing on the incidence of CRBSIs, and the results showed that chlorhexidine dressing significantly reduced the incidence of CRBSIs (RR 0.55, 95% CI 0.39–0.77, *P*<0.001) (Fig. 4.) in hospitalized patients. In addition, we performed a subgroup analysis showing that chlorhexidine dressing significantly reduced the incidence of CRBSIs in both ICU (RR 0.55, 95% CI 0.31–0.97, *P*=0.04) and non-ICU (RR 0.60, 95% CI 0.40–0.90, *P*=0.01).

**Fig. 5:**
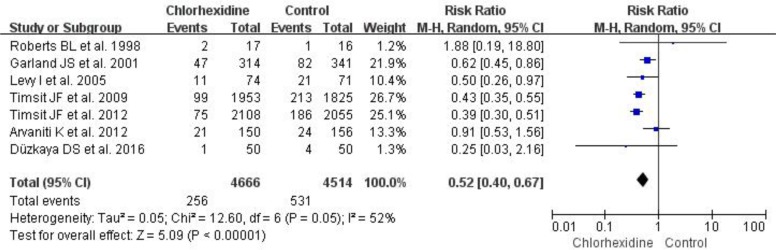
Forest plot of chlorhexidine dressing and control groups on the incidence of catheter colonization using a random-effects model. M-H indicates Mantel-Haenszel

**Fig. 6:**
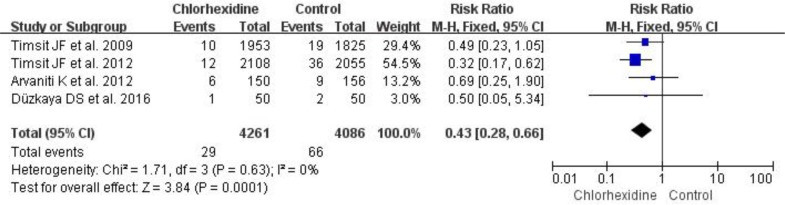
Forest plot of chlorhexidine dressing and control groups on the incidence of catheter-related infections using a fixed-effects model. M-H indicates Mantel-Haenszel

Seven RCTs reported the relationship between chlorhexidine dressing and the incidence of catheter colonization (9, 10, 13–15, 23, 24), and the incidence of catheter colonization was 5.5% (256/4666) in the chlorhexidine group and 11.8% (531/4514) in the control group. Our forest plot results suggested that chlorhexidine dressing significantly reduced the incidence of catheter colonization (RR 0.52, 95% CI 0.40–0.67, *P*<0.001) (Fig. 5.) in hospitalized patients. Moreover, four RCTs reported the effects of chlorhexidine dressing on the incidence of CRIs (10, 13, 15, 24), and the incidence of CRIs was 0.7% (29/4261) in the chlorhexidine group and 1.6% (66/4086) in the control group. Our forest plot results showed that chlorhexidine dressing significantly reduced the incidence of CRIs (RR 0.43, 95% CI 0.28–0.66, *P*<0.001) (Fig. 6.) in hospitalized patients.

### Publication bias

**Fig. 7:**
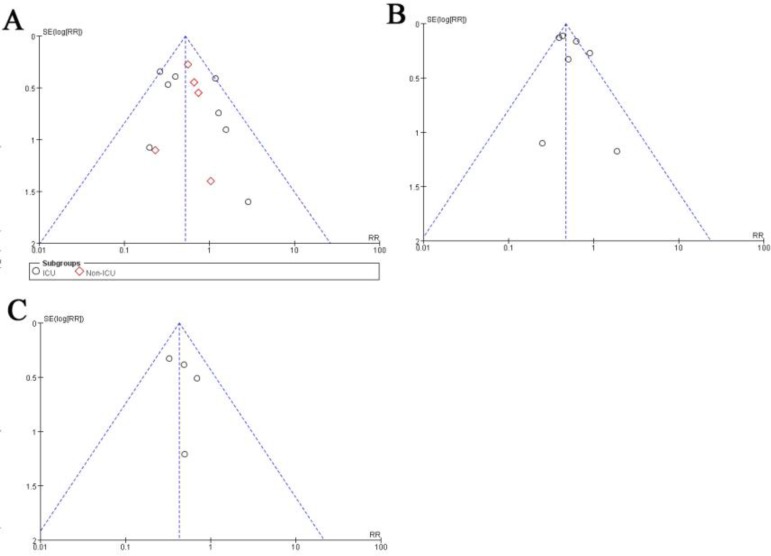
Funnel plots of meta-analysis for the effects of chlorhexidine dressing on catheter-related bloodstream infections (A, Begg’s test, *P*=0.42; Egger’s test, *P*=0.67), catheter colonization (B, Begg’s test, *P*=0.21; Egger’s test, *P*=0.35), and catheter-related infection (C, Begg’s test, *P*=0.46; Egger’s test, *P*=0.90). The results revealed no publication bias, as all *P* values were >0.05. SE, standard error; RR, risk ratio

We used a funnel plot and Begg’s and Egger’s test to assess included RCTs publication bias, and our results showed that the incidence of CRBSIs, catheter colonization and CRIs were no publication biased (*P*>0.05) (Fig. 7.).

## Discussion

Inpatients often need to establish intravascular catheters to treat critically ill and severe diseases such as cancer chemotherapy, parenteral nutrition, hemodialysis, long-term intravenous antibiotics and organ transplantation, etc. (25, 26). In the United States, more than 5 million inpatients require central venous access each year (27). However, catheter-related bloodstream infections (CRBSIs) is an important factor leading to increased hospital stay, total cost, and increased mortality (28). The occurrence of CRBSIs is usually caused by skin microbes invading the subcutaneous pipeline, and blocking the displacement of microorganisms can effectively prevent medically relevant CRBSIs (29). Skin disinfection with chlorhexidine significantly reduce the incidence of CRBSIs, which is simple, effective and cost-effective (30).

A number of studies reported that chlorhexidine dressing can reduce the invasion of extra-catheter microbes and reduce the incidence of CRBSIs (9–13). However, some studies found that the use of chlorhexidine dressing did not have any effect on the incidence of CRBSIs. In our study, we used a meta-analysis to determine the effects of chlorohexidine dressing on the incidence of CRBSIs, catheter colonization and catheter-related infection (CRIs) in hospitalized patients. A total of 13 RCTs were included in our meta-analysis, including 7555 patients and 11,931 catheters. Our results showed that chlorhexidine dressing significantly reduced the incidence of CRBSIs in hospitalized patients. To determine whether chlorhexidine dressings are equally effective in preventing the incidence of CRBSIs in ICU and non-ICU patients, we performed a subgroup analysis. Our results showed that chlorhexidine dressing significantly reduced the incidence of CRBSIs in both ICU and non-ICU. These results indicated that the use of chlorhexidine dressing significantly reduced the invasion of microbes outside the catheter and inhibited the growth of skin microbes (6–8).

The six studies included in our meta-analysis first disinfected the skin with chlorhexidine and then covered the catheter inlet with chlorhexidine dressings (14, 16–18, 23, 24), four studies used alcohol for skin disinfection (9–11, 22), and one study did not record the disinfectant used for skin disinfection (15). Moreover, of the 13 RCTs, six RCTs used chlorhexidine-impregnated sponge dressings (10, 11, 13–15, 23), and seven RCTs used the chlorhexidine dressings (9, 12, 16–18, 22, 24), which did not indicate the type.

A meta-analysis (31), reported that the use of chlorhexidine impregnated dressings can effectively prevent CRBSIs, including arterial catheters for hemodynamic monitoring. In our meta-analysis, eight studies previously evaluated were included (9–11, 14, 15, 22–24), and four RCTs published in recent years were included (12, 13, 16–18), excluding a study that did not retrieve the full text. We also analyzed the relationship between chlorhexidine dressing and the incidence of catheter colonization. Seven RCTs were included in our analysis (9, 10, 13–15, 23, 24), and the incidence of catheter colonization was 5.5% (256/4666) in the chlorhexidine group and 11.8% (531/4514) in the control group. Our results suggested that the use of chlorhexidine dressing significantly reduced the incidence of catheter colonization in hospitalized patients. Moreover, four RCTs reported the effect of chlorhexidine dressings on the incidence of CRIs (10, 13, 15, 24), and our forest plot results showed that chlorhexidine dressing also significantly reduced the incidence of CRIs in hospitalized patients.

Our meta-analysis has four limitations. Firstly, the main research object of most of the studies we have included were central venous catheters (CVCs), but one study was peripherally inserted central catheters (PICCs). Different methods of indwelling CVCs might have an impact on the results of the study. Secondly, we only included full-text journal articles published in English, and non-English languages and conference papers were excluded. Therefore, some RCTs were not included in our analysis, which might lead to publication bias or heterogeneity. Thirdly, the products of chlorhexidine dressing used in the studies were different, and the doses of chlorhexidine contained in the dressings were also different. These factors might have a negative impact on these studies. Fourthly, the effectiveness of chlorhexidine dressings for CRBSI prevention might be inconsistent among different populations, such as neonates, children, adults and seniors. However, our analysis did not separate these populations, so our results might be heterogeneous.

## Conclusion

The use of chlorhexidine dressings significantly reduced the incidence of CRBSIs, catheter colonization and CRIs in hospitalized patients. Our results support the use of chlorhexidine dressings in hospitalized patients with indwelling CVCs, which has important implications for CVCs care. Future research should focus on which populations may benefit the most from the use of chlorhexidine dressings, the frequency of chlorhexidine dressing replacement, and the longest indwelling time of CVCs.

## Ethical considerations

Ethical issues (Including plagiarism, informed consent, misconduct, data fabrication and/or falsification, double publication and/or submission, redundancy, etc.) have been completely observed by the authors.
